# Transcriptome Data Combined With Mendelian Randomization Analysis Identifies Key Genes Associated With Mitochondria and Programmed Cell Death in Intervertebral Disc Degeneration

**DOI:** 10.1002/jsp2.70057

**Published:** 2025-03-24

**Authors:** Hongfei Nie, Xiao Hu, Jiaxiao Wang, Jia Wang, Xiaoqian Yu, Jun Li

**Affiliations:** ^1^ Department of Pain Management, West China Hospital Sichuan University Chengdu Sichuan Province China; ^2^ Frontiers Science Center for Disease‐Related Molecular Network, Department of Orthopedic Surgery and Orthopedic Research Institute, West China Hospital Sichuan University Chengdu Sichuan Province China

**Keywords:** intervertebral disc degeneration, key genes, mitochondria‐related genes, programmed cell death‐related genes

## Abstract

**Background:**

Intervertebral disc degeneration (IDD) is a major cause of cervical and lumbar diseases, significantly impacting patients' quality of life. Mitochondria and cell death have been implicated in IDD, but the key related genes remain unknown.

**Methods:**

Differentially expressed genes (DEGs) between IDD and control samples were identified using GSE70362. Mitochondria‐related genes (MRGs) and programmed cell death‐related genes (PCDRGs) were intersected with DEGs to find DE‐MRGs and DE‐PCDRGs. Weighted gene co‐expression network analysis (WGCNA) identified key module genes, and the overlap with DEGs revealed candidate genes. Mendelian randomization (MR) analysis was used to determine genes causally linked to IDD. Machine learning and expression validation further refined key genes, which were then used to build a nomogram to predict IDD risk. Additionally, gene set enrichment analysis (GSEA), immune infiltration, and single‐cell analysis were performed.

**Results:**

A total of 515 DEGs were intersected with 224 key module genes, yielding 31 candidate genes. Six genes—BCKDHB, BID, TNFAIP6, VRK1, CAB39L, and TMTC1—showed a causal relationship with IDD. BID, TNFAIP6, and TMTC1 were further identified as key genes through machine learning and validation. A nomogram was developed based on these genes. GSEA revealed BID and TMTC1 were enriched in N‐glycan biosynthesis, TNFAIP6 and TMTC1 in aminoacyl tRNA biosynthesis, and BID and TMTC1 in ribosomal pathways. Activated dendritic cells, CD56dim natural killer cells, monocytes, and other immune cells were elevated in IDD, with TNFAIP6 strongly correlating with activated dendritic cells. Key genes were expressed at higher levels in degraded samples.

**Conclusion:**

BID, TMTC1, and TNFAIP6 were identified as key genes linked to mitochondria and cell death in IDD, offering new insights for diagnosis and treatment.

## Graphical abstract

This research underlines the translational potential of our findings in IDD, focusing on mitochondrial dysfunction and programmed cell death pathways. By pinpointing genes like BID, TMTC1, and TNFAIP6, our work offers promising targets for therapeutic development and diagnostic markers. The construction of predictive nomograms based on these genes could facilitate early diagnosis and personalized treatment strategies, significantly enhancing clinical outcomes for patients with IDD.

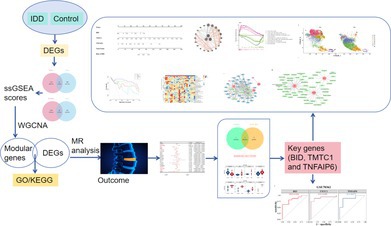

## 1 Introduction

Intervertebral disc degeneration (IDD) is a condition characterized by the gradual deterioration of intervertebral discs, leading to structural and functional abnormalities [1, 2]. It is a primary cause of neck and lower back pain [3]. Anatomically, the disc's anatomy includes the annulus fibrosus, cartilage endplates, and nucleus pulposus, with limited blood supply hindering repair after damage [4, 5]. The three main pathophysiological changes in nucleus pulposus degeneration are the metabolic imbalance of the extracellular matrix (ECM), apoptosis, and inflammation [6]. Factors such as smoking, overweigh [7], biomechanical stress [8], genetic predisposition [9], and cellular metabolic issues contribute to the progression of IDD [10, 11]. Although there is some understanding of the etiology and pathology of IDD, the insufficient knowledge of its biological regulation hampers the development of effective diagnosis and treatment strategies [12]. The pain associated with IDD leads to a decline in quality of life and high medical expenses, imposing a significant burden on both individuals and society [13]. Conservative treatments and necessary surgical interventions often fail to fundamentally address the disc pathology, with postoperative complications such as nerve damage and spinal pain further limiting their clinical outcomes [14]. Therefore, an in‐depth understanding of IDD's pathophysiological mechanisms is crucial for early and proactive intervention in disease progression and reducing the rate of surgeries.

Programmed cell death (PCD), controlled by genetic mechanisms, includes various types such as necroptosis, apoptosis, autophagic cell death, and more recently discovered types like ferroptosis and cuproptosis [15, 16]. Mitochondria, as cellular organelles involved in energy metabolism, have gained increasing attention in recent years for their roles in various diseases. Mitochondrial dysfunction can lead to the onset of various pathological mechanisms, including the loss of mitochondrial membrane potential, decreased oxidative phosphorylation capacity, and the release of pro‐apoptotic factors [17, 18]. Mitochondria not only provide the primary energy supply for cells but also act as central regulators of apoptosis, having been proven to regulate cell death through multiple pathways [19]. In the context of IDD, the nucleus pulposus relies on its ECM to alleviate stress, and the metabolic imbalance of the ECM is associated with PCD [20, 21, 22]. Mitochondrial dysfunction allows PCD, oxidative stress, and mitochondrial autophagy to crosstalk with each other, causing intervertebral disc cell death and disruption of ECM homeostasis, which ultimately contributes to the onset and progression of IDD [23, 24, 25]. Prior research has proposed that mitochondrial dysfunction and PCD might be pivotal in IDD's pathogenesis [26]. However, the potential causal relationship between PCD and mitochondrial dysfunction in IDD remains unclear due to the confounding factors inherent in past research methodologies such as RCTs and the compensatory mechanisms observed in animal studies. Therefore, it is crucial to find a systematic and in‐depth research approach.

Transcriptomics aims to investigate the total transcription products of all genes within an organism. This method can offer more comprehensive gene expression information, aiding in the identification of potential biological processes and disease mechanisms, as well as the discovery of new biomarkers [27]. Single‐cell technology allows for the analysis of target gene expression at the level of individual cells, aiding in the revelation of cellular states and identification of different cell subpopulations [28]. Its application in IDD research facilitates a deeper exploration of potential disease mechanisms. Mendelian randomization (MR) is a potent statistical method aimed at identifying causal linkages between exposure variables and targeted diseases [29, 30]. This method integrates the principles of Mendel's laws, which state that alleles assort randomly during sexual reproduction, thus combining in offspring in an unbiased manner [31]. Consequently, MR offers a distinct advantage over RCTs by adeptly bypassing their constraints. It significantly reduces confounding effects and minimizes the risk of reverse causality to the maximum extent [32]. To date, MR has been applied in studies to explore potential exposure factors for IDD [33, 34]; however, there is a paucity of research delving into the roles of mitochondrial and cell death pathways in IDD.

In this study, we employed transcriptomic data combined with MR to screen and identify key differentially expressed programmed cell death‐related genes (DE‐PCDRGs) and differentially expressed mitochondria‐related genes (DE‐MRGs) in IDD. We analyzed the biological pathways in which these key genes participate investigated their roles in molecular regulatory networks, and related pharmaceuticals. Additionally, we observed the distribution and expression of these key genes in a single‐cell dataset, providing new theoretical support and reference for disease treatment.

## 2 Materials and Methods

### 2.1 Data Acquisition

IDD‐related datasets were obtained from the Gene Expression Omnibus (GEO) database (https://www.ncbi.nlm.nih.gov/geo/). The GSE70362, as a training set, contained transcriptome microarray sequencing data from nucleus pulposus tissue samples of 16 IDD and eight control samples on the GPL17810 sequencing platform, according to Thompson Grade, the samples of grade I, II, and I‐II were used as control samples, and the samples of grade III, IV, and V were used as IDD samples. GSE56081 (platform: GPL15314) was used as validation set, which contained nucleus pulposus tissue samples of five IDD and five controls. In addition, IDD single‐cell dataset GSE199866 was selected for follow‐up single‐cell analysis, in which samples of degraded and non‐degraded intervertebral disc nucleus pulposus cells were screened, and one sample was retained respectively for follow‐up analysis. In all 1136 MRGs were obtained from the MitoCarta 3.0 database (https://www.broadinstitute.org/mitocarta), and 1548 PCDRGs were obtained from previous studies [35]. IDD‐related Genome‐wide association study (GWAS) dataset (ukb‐b‐19 807) was obtained from Integrative Epidemiology Unit (IEU) OpenGWAS database (https://gwas.mrcieu.ac.uk) by searching “specified intervertebral disk”, including 4690 cases and 356 504 controls (all European populations), and 12 016 668 single‐nucleotide polymorphisms (SNPs).

### 2.2 Differential Analysis and Weighted Gene Co‐Expression Network Analysis (WGCNA)

Differential analysis was applied to gain differentially expressed genes (DEGs) between IDD and control in GSE70362 using limma (v3.54.0) [36], with *p* value < 0.05 and |log_2_FC| > 0.5. The Top10 up‐regulated and down‐regulated DEGs were shown by volcano map and heat map using ggplot2 (v3.4.1) [37] and ComplexHeatmap (v2.14.0) [38] respectively. DEGs were overlapped with MRGs and PCDRGs, respectively, to obtain DE‐MRGs and DE‐PCDRGs.

Subsequently, to obtain the module genes associated with DE‐MRGs and DE‐PCDRGs, WGCNA (v1.71) [39] was employed for WGCNA. Firstly, the correlation scores of DE‐MRGs and DE‐PCDRGs in GSE70362 samples were calculated by single sample Gene Set Enrichment Analysis (ssGSEA) through GSVA (v1.46.0) [40], and the differences in these scores between IDD and control were analyzed (*p* < 0.05). Then, these scores were used as traits for WGCNA. All samples in the training set were clustered, and outliers were removed. Then, based on *R* ^2^ = 0.85, the soft threshold was determined. Next, the genes were divided into multiple modules according to dynamic tree cutting (minModuleSize =100, deepSplit = 4, mergeCutHeight = 0.4). Afterward, the module with higher correlation with the two traits was selected as the key module (|*r*| > 0.5 and *p* < 0.05). Eventually, the genes in key modules were screened according to module membership (MM) > 0.7 and gene significance (GS) > 0.3 as mitochondrial and cell death‐related module genes and considered as key module genes.

### 2.3 Identification and Enrichment Analysis of Candidate Genes

The candidate genes were obtained via overlap of DEGs and key module genes. Venn diagrams were plotted using ggvenn (v0.1.9) [41] for presentation. Further, enrichment analysis was utilized to probe functions and pathways involved in the candidate genes using clusterProfiler (v4.2.2) [42], including Gene Ontology (GO) and Kyoto Encyclopedia of Genes and Genomes (KEGG) (*p* < 0.05).

### 2.4 MR Analysis and Functional Analysis

Candidate genes were used as exposure factors and IDD as the outcome for MR analysis. MR studies must satisfy the following three assumptions: (1) there is a strong correlation between instrumental variables (IVs) and exposure, (2) IVs are independent of confounding factors that are related to exposure and outcome, and (3) IVs affect the outcome only by exposure and not other biological pathways. The ld_clump of ieugwasr and format_data of TwoSampleMR (v0.5.7) [43] were used to find the IVs that significant correlation with the exposure factors and unrelated to the outcome were selected for MR analysis. Then, for the linkage disequilibrium (LD) study, IVs were clumped (*r* ^2^ = 0.001 and kb = 10). Importantly, when the F‐statistic value was less than 10, it meant that the IVs were weak and eliminated. In addition, the exposure factors with fewer than three SNPs were also eliminated.

The TwoSampleMR function harmonise_data was used to unify the effect alleles and effect sizes, and MR analyses were performed by mr in combination with five algorithms, including MR Egger [44], Weighted median [45], Inverse variance weighted (IVW) [46], Simple mode [47], and Weighted mode [48], which focused on the results of the IVW analysis. An odds ratio (OR) value greater than 1 is a risk factor, and less than 1 is a protective factor. The results were presented through scatter plots, forest plots, and funnel plots. Then, Steiger was used to test the directionality of the MR analysis. Subsequently, the reliability of the MR analysis was tested by sensitivity analysis, in which the heterogeneity analysis was executed using the mr_heterogeneity function; the I2 index and Cochran's Q statistic were adopted for the IVW analysis. If *p* > 0.05, it indicated that there was no heterogeneity between the two datasets [49]. Then, Horizontal pleiotropy tests [50] were conducted by the TwoSampleMR function mr_pleiotropy_test and the MRPRESSO function mr_presso, which, if *p* > 0.05, indicated that there were no confounders. The leave‐one‐out (LOO) [51] was implemented via the mr_leaveoneout function and used to see if there were outliers in the effect of each SNP. Genes with a causal relationship with IDD were used as a key exposure factor for subsequent analysis.

In addition, the functional similarity of key exposure factors was evaluated using GOSemSim (v2.24.0) [52], and then Spearman was used to calculate the correlation between key exposure factors using corrplot (v0.92) [53].

### 2.5 Machine Learning and Expression Verification

To further screen for key genes of IDD, we performed machine learning. Initially, least absolute shrinkage and selection operator (LASSO) analysis was performed using glmnet (v4.1‐4) [54], and the best lambda value was selected after 5‐fold cross‐validation to screen the feature genes. Meanwhile, caret (v6.0‐93) [55] was used to perform support vector machine recursive feature elimination (SVM‐RFE) analysis to screen key exposure factors, and the gene with the highest prediction accuracy was selected as the characteristic gene. Then, intersection genes were obtained via the overlap of two machine learning results. Eventually, the expression profiles of these genes were extracted in the training and validation sets, selecting as key genes those genes whose expression trends were consistent in both datasets and which differed markedly between disease and control.

### 2.6 Diagnostic Analysis of Key Genes and Construction of Nomogram

Further, in order to explore the diagnostic value of key genes for IDD, receiver operating characteristic (ROC) curves of key genes were constructed with pROC (v1.18.0) [56] and area under the curve (AUC) values were calculated. When the AUC value is greater than 0.7, it indicates that this gene has better diagnostic efficacy for IDD. Afterward, based on key genes, rms (v6.5‐0) [57] was used to draw a nomogram of predictive diagnostics to predict IDD risk. Both ROC curve, calibration curve, decision curve analysis (DCA), and clinical impact curve (CIC) were used to examine the accuracy of thenomogram.

### 2.7 Functional and Pathway Analysis of Key Genes

In the training set, the correlation coefficients of each key gene and other genes were calculated by Spearman, sorted from the largest to the smallest, and then GSEA was conducted based on the KEGG gene set in msigdbr (v7.5.1) [58] as the background gene set. The top 5 signaling pathways enriched by key genes with the largest and smallest NES were visualized using enrichplot (v1.18.0) [59]. Further, the co‐expression networks and biological functions of key genes were analyzed using the GeneMANIA database (http://genemania.org/).

### 2.8 Immune Infiltration Analysis

In order to explore the role of key genes in the IDD immune microenvironment, the enrichment scores of 28 kinds of immune infiltrating cells in the training set samples were calculated based on ssGSEA. Then Wilcoxon was used to compare the concentration of immune infiltrating cells between IDD and control. After that, the correlation between differential immune cells, as well as differential immune cells and key genes was analyzed.

### 2.9 Regulatory Mechanisms and Drug Prediction

In order to understand the role of key genes in IDD, the transcription factors corresponding to key genes were predicted through the JASPER database (http://jaspar.genereg.net/) and ChIP‐X Enrichment Analysis (ChEA) database (Maayanlab.cloud/chea3), and then the two results were intersected to obtain transcription factors (TF)‐key gene relationship pairs. In addition, the target miRNAs of key genes were predicted by TarBase (http://www.microrna.gr/tarbase) and miRTarBase (http://miRTarBase.mbc.nctu.edu.tw/) databases, and then the results were overlapped to obtain the intersection miRNA‐mRNA. Then the StarBase database (http://starbase.sysu.edu.cn) was used to predict lncRNAs corresponding to targeted miRNAs, and then miRNAs co‐regulated by key genes and lncRNAs were screened. An lncRNA‐miRNA‐mRNA network was constructed. Moreover, the Comparative Toxicogenomics Database (CTD) (https://ctdbase.org/) was used to predict the chemical agents corresponding to key genes.

### 2.10 Single‐Cell RNA‐Sequencing (scRNA‐Seq) Analysis

To further analyze the expression of key genes in single cells, we performed single‐cell analysis in GSE199866. Originally, cells with less than 200 genes and genes covered by less than three cells in the dataset were first filtered using Seurat (v4.3.0) [60]. The number of genes measured per cell (nFeature_RNA, greater than 500 and less than 5.000), the sum of the expression of all genes measured in each cell (nCount_RNA, less than 30 000), and the percentage of mitochondrial genes measured per cell (percent.mt) less than 5% were used as quality control conditions. Subsequently, the FindIntegrationAnchors function was used to identify anchors, and the identified anchors were applied to integrate the dataset through the IntegrateData function to remove batch effects. Then, the data was normalized using the NormalizeData function, and the FindVariableFeatures function was utilized to select the first 2000 highly variable genes for subsequent analysis. The ScaleData function was applied for data normalization. The resolution of cell group identification was set to 0.6, and uniform manifold approximation and projection (UMAP) cell cluster analysis was performed. Furthermore, the cells in GSE199866 were identified as nucleus pulposus cells according to previous studies [61, 62], and the marker genes between the two samples were identified in nucleus pulposus cells using the FindAllMarkers function (*p*_val_adj < 0.05&|avg_log_2_FC| > 0.25, min.pct = 0.25). Next, the expression of key genes in nucleus pulposus cells was analyzed. Finally, monocle (v2.26.0) [63] was used to simulate the cell trajectory of nucleus pulposus cells.

### 2.11 Statistical Analysis

All analyses were conducted using the R language (v4.1.0), and the data from different groups were compared by the Wilcoxon test. If not specified above, a *p*‐value less than 0.05 was considered statistically significant.

## 3 Results

### 3.1 Identification of 224 Key Module Genes

**FIGURE 1:**
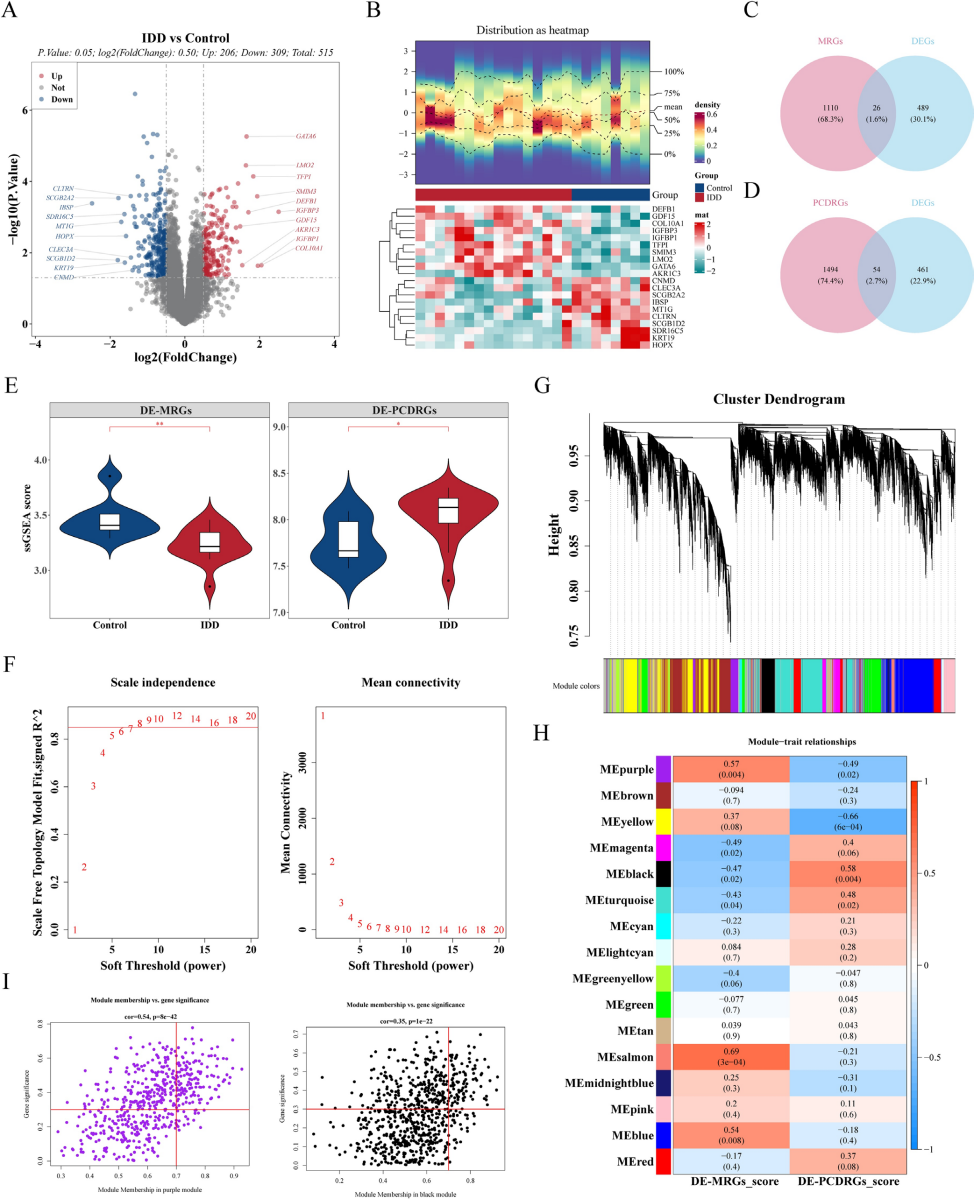
Identification of differential expression genes (DEGs) between intervertebral disc degeneration (IDD) and control samples as well as key module genes within GSE70362 dataset. (A) Volcano plot of DEGs, with red dots signifying up‐regulated genes, blue dots representing down‐regulated genes, and gray dots indicating undifferentiated genes. (B) Heat map of top 10 up‐ and down‐regulated DEGs. (C) Venn diagram illustrates the differentially expressed mitochondria‐related genes (DE‐MRGs) by overlapping DEGs and MRGs. (D) Venn diagram illustrates the differentially expressed programmed cell death‐related genes (DE‐PCDRGs) by overlapping DEGs and PCDRGs. (E) The single sample Gene Set Enrichment Analysis (ssGSEA) scores of DE‐MRGs and DE‐PCDRGs. (F) Analysis of the scale‐free index and mean connectivity for various soft threshold powers. (G) The gene dendrogram is generated using average linkage hierarchical clustering. The module assignment determined by the Dynamic Tree Cut algorithm is displayed below the dendrogram. (H) Positive and negative correlation coefficients of the weighted gene co‐expression network analysis (WGCNA) modules among the scores of DE‐MRGs and DE‐PCDRGs. Red indicated a positive correlation, blue a negative correlation; numbers in the top half of the cell indicated correlation, numbers in parentheses indicated significance. (I) The module membership (MM) and gene significance (GS) of key module genes. *p < 0.05, **p < 0.01.

The 515 DEGs were gained between IDD and control, which contained 309 low‐expressed DEGs and 206 high‐expressed DEGs (Figure 1A,B). Then, 26 DE‐MRGs and 54 DE‐PCDRGs were received (Figure 1C,D). Subsequently, the scores of DE‐MRGs and DE‐PCDRGs in the training set samples were calculated by ssGSEA, and they were found to be remarkably different between both IDD and control (Figure 1E). Afterward, the cluster analysis of the training set samples found that GSM1725809 was an obvious outlier and eliminated (Figure S1). *R* ^2^ was set to 0.85, and the soft threshold β was screened to eight (Figure 1F) to build a scale‐free network. A total of 16 co‐expression modules were identified by WGCNA (Figure 1G), among which purple modules and black modules were highly correlated with the two traits (Figure 1H). After screening, 224 mitochondrial and cell death‐related module genes were obtained as key module genes (Figure 1I).

### 3.2 Identification and Function Enrichment of 31 Candidate Genes

**FIGURE 2:**
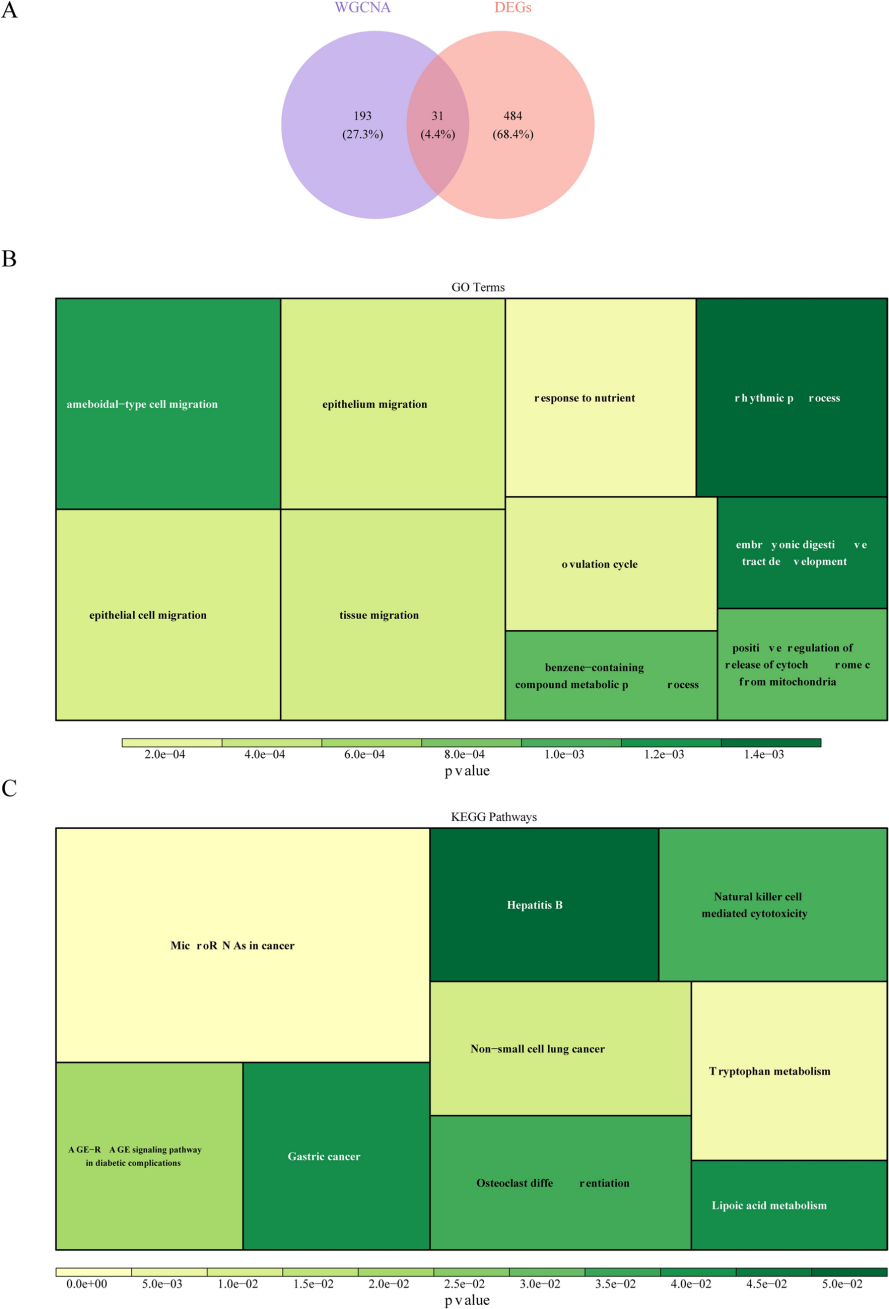
Identification and enrichment analysis of candidate genes. (A) Venn diagram illustrates the candidate gene by overlapping DEGs and key module genes. (B) Gene Ontology (GO) term of candidate genes. The area of the square represents the size of the count (number of genes), the larger the area, the larger the count. (C) Kyoto Encyclopedia of Genes and Genomes (KEGG) pathway of candidate genes. The area of the square represents the size of the count (number of genes), the larger the area, the larger the count.

The 31 candidate genes were screened via the intersection of DEGs and key module genes (Figure 2A). Then, GO results indicated that these genes were involved in response to nutrients, epithelium migration, and tissue migration (Figure 2B). KEGG results showed that candidate genes were mainly markedly enriched in MicroRNAs in cancer, Tryptophan metabolism, and AGE‐RAGE signaling pathway in diabetic complications (Figure 2C).

### 3.3 Screening and Function Analysis of Six Key Exposure Factors

**FIGURE 3:**
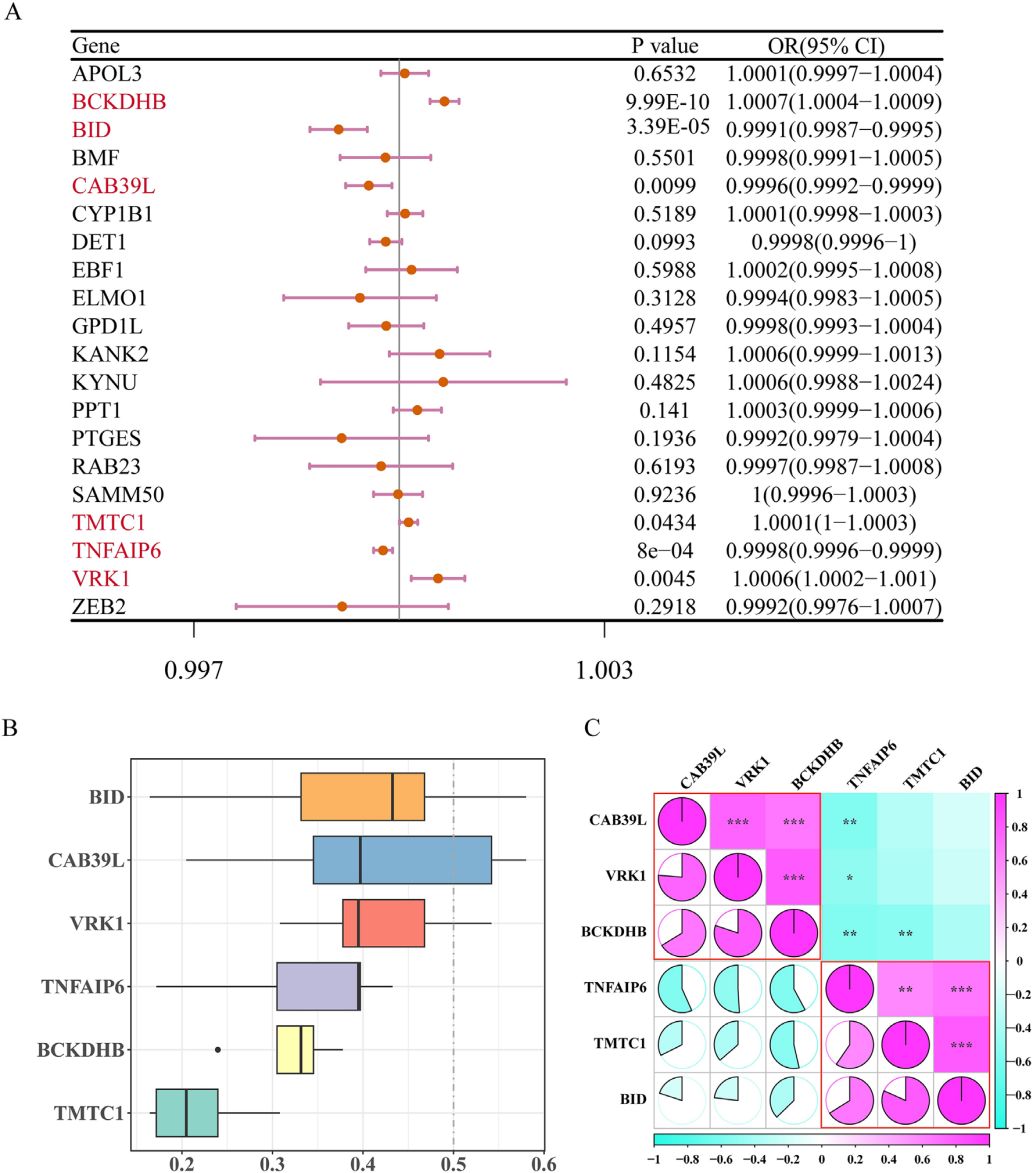
Mendelian randomization (MR) analysis probing key exposure factors. (A) Forest plot displaying the results of MR analysis of causal associations between candidate genes and IDD. (B) Functional similarity analysis of key exposure factors. The *X*‐axis represented the mean of the similarity scores. (C) Analysis of correlations among key exposure factors. The *X*‐axis represented the correlation coefficient.

After removing the exposure factor with SNP below three, 20 candidate genes were used as the exposure factor, and IDD was used as the outcome for MR analysis. IVW results showed that *p* values of six genes were all less than 0.05, indicating that they had a significant causal relationship with IDD. Among them, the OR values of BCKDHB, VRK1, and TMTC1 were greater than 1, the scatter plot showed that the slope of IVW was positive, and the intercept was close to 0 (Figure S2), and the forest plot showed that the overall effect of IVW was greater than 0 (Figure S3), indicating that they were risk factors for IDD. While the OR values of TNFAIP6, CAB39L, and BID were less than 1, the scatter plot showed that the slope of IVW was negative, and the intercept was close to 0 (Figure S2), and the forest plot showed that the overall effect of IVW was less than 0 (Figure S3), indicating that they were protective factors of IDD (Figure 3A). Then, they all passed the directionality test (Table S1). In addition, the funnel diagram shows that the number of SNPs was randomly and evenly distributed on both sides of IVW, which accorded with Mendel's second law (Figure S4).

Further, the reliability of MR Analysis was verified by sensitivity analysis. Heterogeneity test showed Q‐*p*val > 0.05, indicating that there was no heterogeneity among data sets (Table S2), and *p*‐value > 0.05 of the horizontal pleiotropy test proved that there were no confounding factors (Table S3). Moreover, the LOO test found a stepwise elimination of each SNP and no significant effect of the remaining SNPs on the outcome (Figure S5). Collectively, this indicates that the results of the MR analysis are reliable and well‐stabilized. Therefore, BCKDHB, VRK1, TMTC1, TNFAIP6, CAB39L, and BID were used as key exposure factors for subsequent analysis.

The functional similarity of the key exposure factors was further assessed and found that BCKDHB, VRK1, and CAB39L had high mean functional similarity (Figure 3B). Additionally, BCKDHB, VRK1, and CAB39L showed positive correlation with each other, while BID, TNFAIP6, and TMTC1 showed positive correlation with each other (Figure 3C).

### 3.4 BID, TMTC1 and TNFAIP6 Were Key Genes of IDD

**FIGURE 4:**
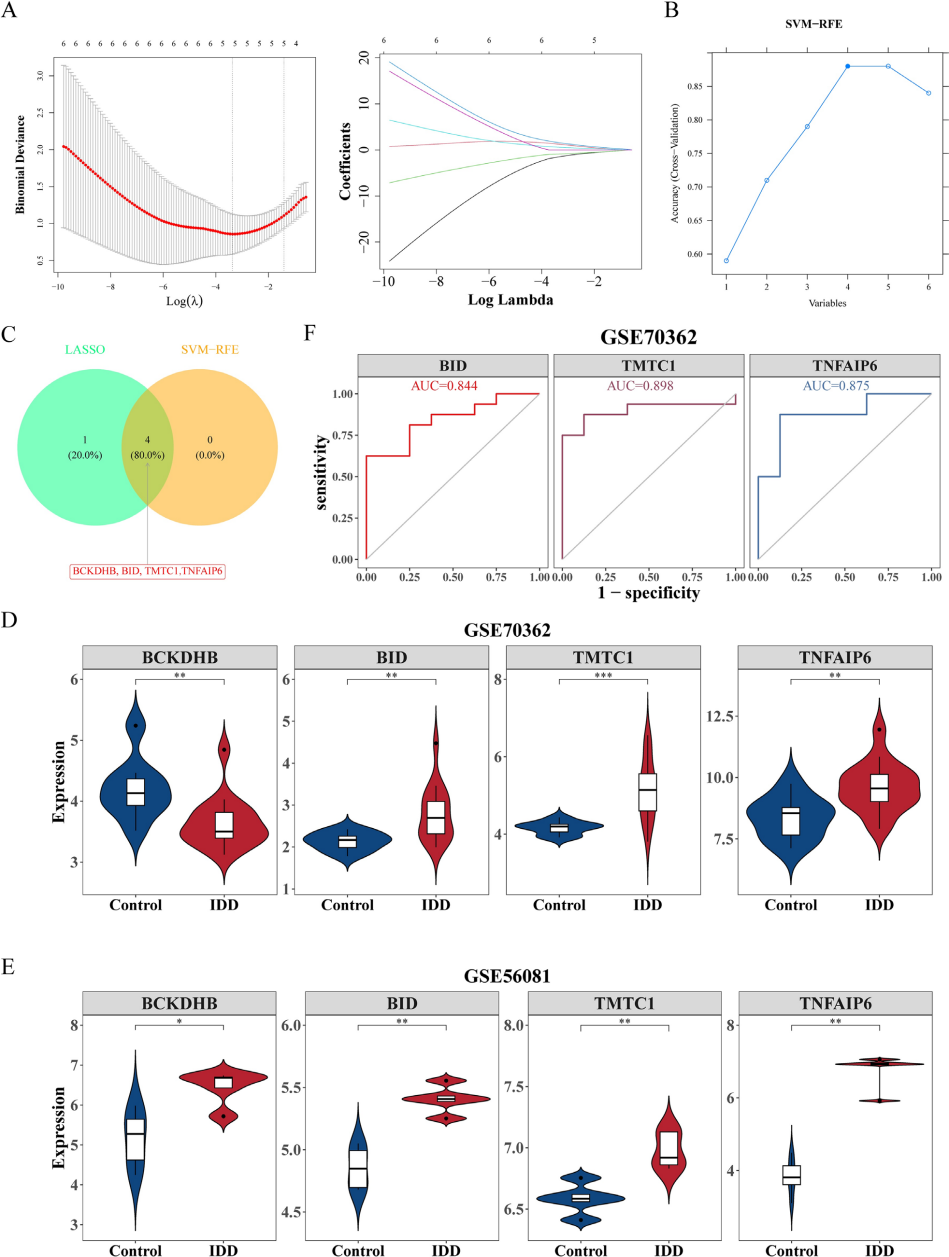
Screening of key genes using machine learning algorithms and expression level analysis. (A and B) Least absolute shrinkage and selection operator (LASSO) and support vector machine recursive feature elimination (SVM‐RFE) to identify characterized genes in dataset GSE70362. (C) Intersection of results obtained from both machine learning algorithms. (D, E) Analysis of expression levels of key genes in IDD versus control samples across datasets GSE70362 and GSE56081. (F) Receiver operating characteristic (ROC) curve analysis for key genes in GSE70362. *p < 0.05, **p < 0.01.

LASSO and SVM‐RFE were employed to screen for five characterized genes (Figure 4A) and four characterized genes (Figure 4B), respectively. Then, four intersection genes were obtained via overlap of these characterized genes, including BCKDHB, TMTC1, TNFAIP6, and BID (Figure 4C). Further, BID, TMTC1, and TNFAIP6 were significantly highly expressed in the IDD group in both the training and validation sets (Figure 4D,E), and the expression trends were consistent. Therefore, they were taken as key genes in this study. In addition, the diagnostic value of the key genes for IDD was tested, and the AUC values of the three key genes were all greater than 0.8, indicating that they had certain diagnostic value for IDD (Figure 4F).

### 3.5 Construction and Validation of Efficient Nomogram

**FIGURE 5:**
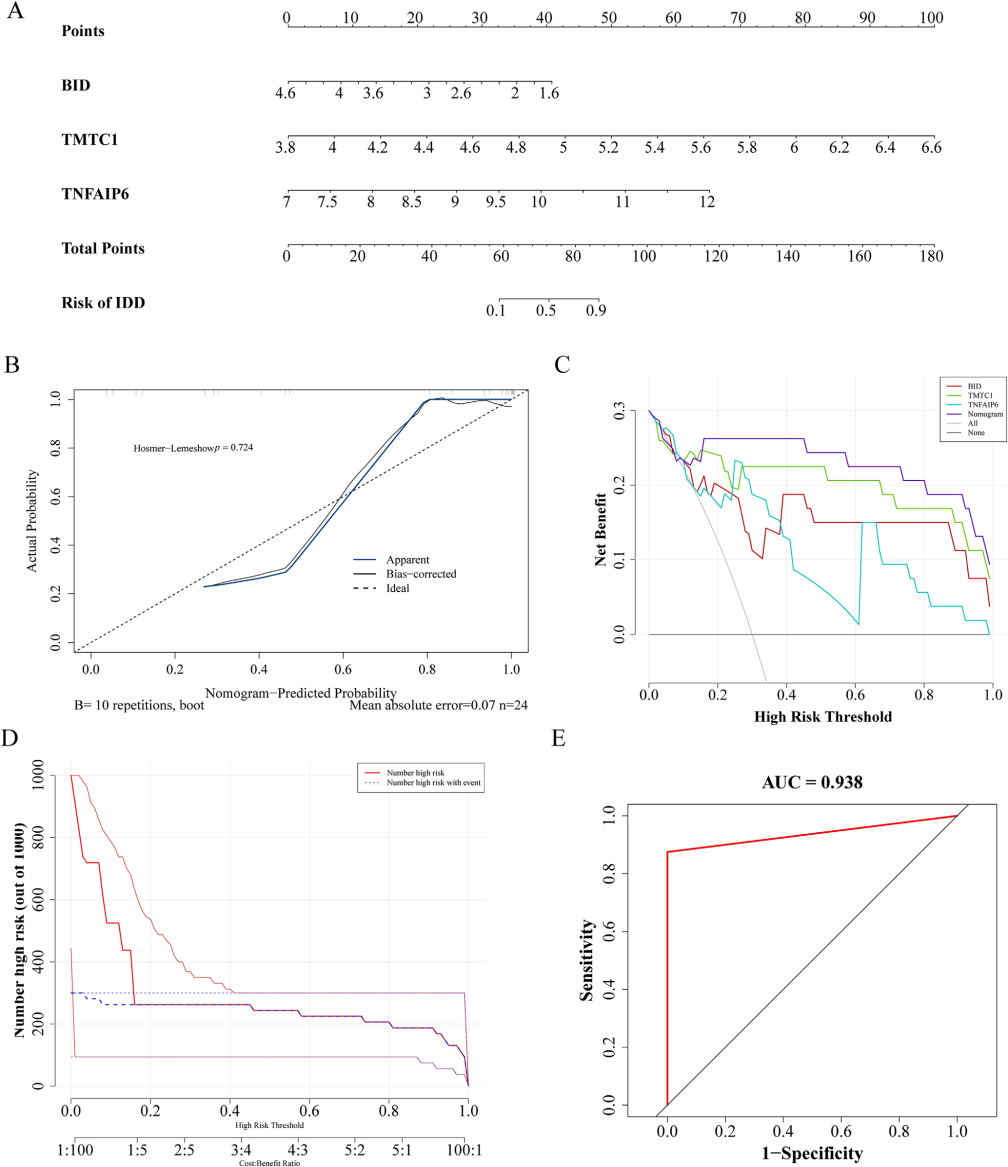
Development of a diagnostic nomogram based on key gene expression in the GSE70362 dataset. (A) Nomogram for assessing the risk of IDD. (B) Calibration curve of the nomogram. (C) Decision curve analysis (DCA) of the nomogram. (D) Clinical impact curve (CIC) of the nomogram. (E) ROC curve of thenomogram.

The nomogram containing three key genes was constructed to predict the risk of IDD (Figure 5A). Furthermore, the *p*‐value of the Hosmer‐Lemeshow (HL) test was 0.724, indicating that there was no significant difference between the predicted value and the true value, which also indicated that the nomogram had high accuracy in predicting IDD (Figure 5B). DCA showed that the nomogram had good benefits (Figure 5C), and CIC proved that the nomogram had clinical validity (Figure 5D). Similarly, the ROC curve (AUC = 0.938) showed that the nomogram had high diagnostic value for IDD (Figure 5E).

### 3.6 Key Genes Were Involved in Complex Functions and Pathways

**FIGURE 6:**
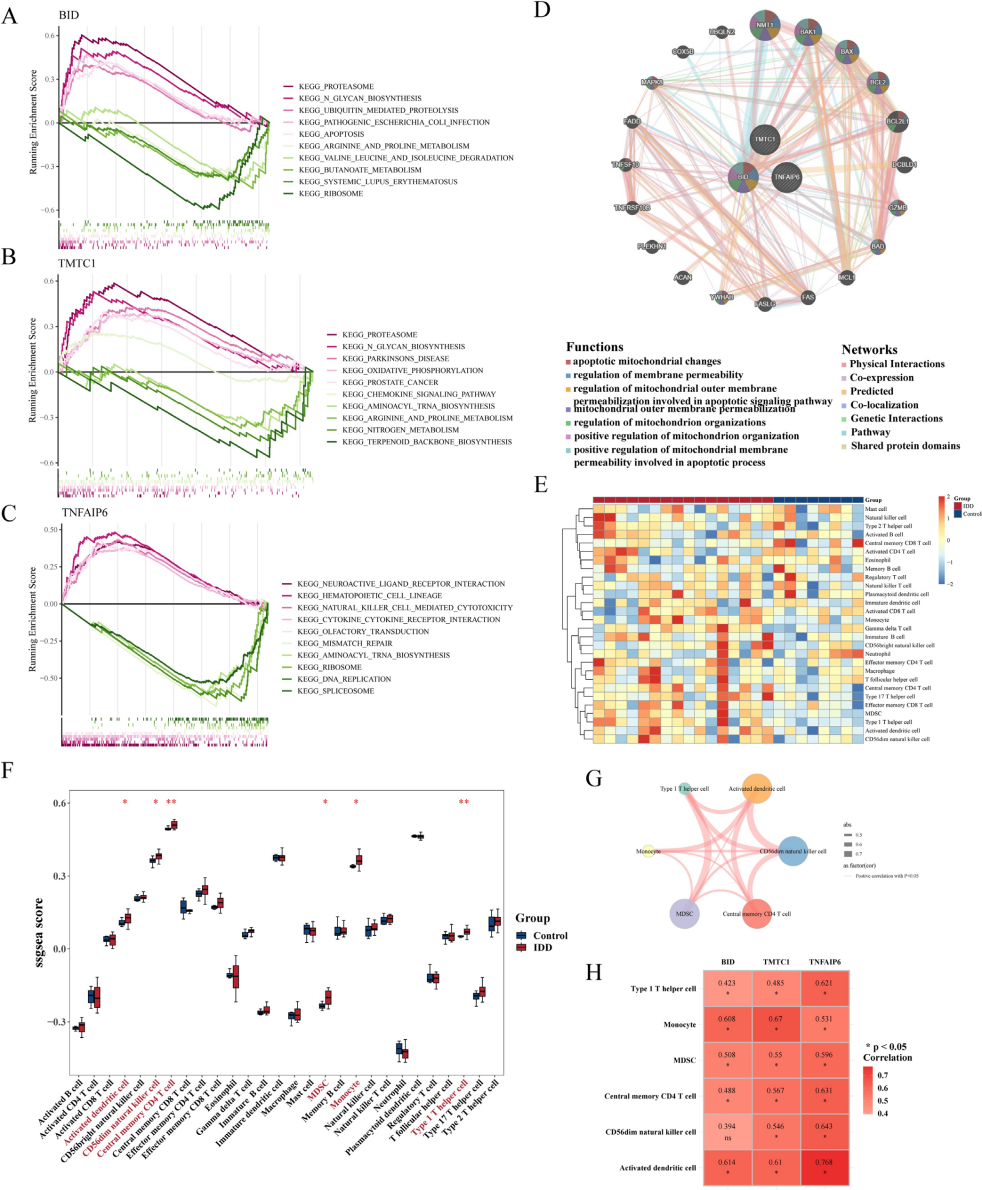
Functional annotation and immune infiltration analyses. (A–C) GSEA of BID, TMTC1, and TNFAIP6 in the GSE70362 dataset. (D) Co‐expression network of key genes constructed leveraging the GeneMANIA database. (E) Abundance of 28 types of immune cell infiltrates between IDD and control samples in the GSE70362 dataset, calculated applying the ssGSEA algorithm. (F) Box plot visualizing differences in immune cell infiltrates between IDD and control samples. (G) Correlation analysis of differential immune cells. (H) Heatmap showing the correlations between differential immune cells and key genes. *p < 0.05, **p < 0.01.

To explore the functions and pathways in which the key genes are specifically involved, we performed GSEA, in which both BID and TMTC1 were enriched for proteasome, N‐glycan biosynthesis, and arginine and proline metabolism pathways. TNFAIP6 and TMTC1 were enriched for aminoacyl TRNA biosynthesis, whereas BID and TNFAIP6 were enriched for ribosomal (Figure 6A–C). Subsequently, the co‐expression network and biological functions of the key genes were analyzed by the GeneMANIA database, in which BID and NMTI, BAK1, BAX, and BCL2 were involved in the processes of mitochondrial outer membrane permeability, regulation of mitochondrial organization, and positive regulation of mitochondrial organization (Figure 6D).

### 3.7 Key Genes Played a Role in the IDD Immune Microenvironment

We analyzed the role of key genes in the immune microenvironment of IDD. Firstly, Figure 6E displayed 28 immune infiltrating cell enrichment scores, and then activated dendritic cell, CD56dim natural killer cell, MDSC, monocyte, central memory CD4 T cell, and type 1 T helper cell were found to be significantly higher in the IDD group by Wilcoxon (Figure 6F). Interestingly, they all showed significant positive correlations with each other (Figure 6G). Moreover, all of the key genes showed positive correlations with differential immune cells, with TNFAIP6 showing the strongest positive correlation with activated dendritic cell (Figure 6H), suggesting a positive correlation between the expression level of this gene and the activity of activated dendritic cell.

### 3.8 The Complex Regulatory Mechanisms of Key Genes and the Prediction of Multiple Drugs

**FIGURE 7:**
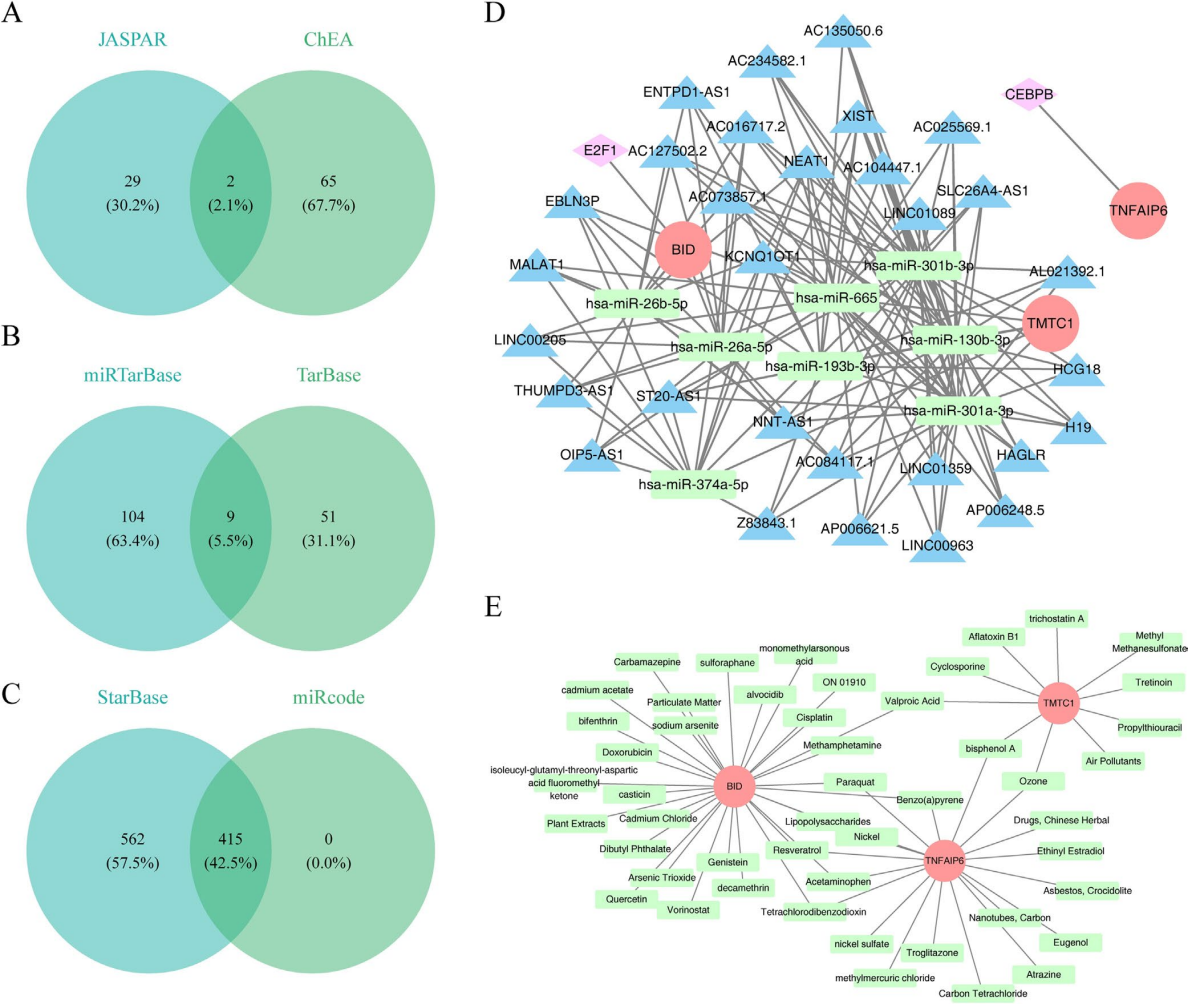
Predictive analysis of key gene regulators Using public databases. (A–C) Identification of potential transcription factors (TFs), microRNAs (miRNAs), and long non‐coding RNAs (lncRNAs) regulating key genes, with Venn diagrams illustrating the intersection of predicted results from respective databases. (D) Construction of a TF‐mRNA regulatory network. (E) Establishment of an lncRNA‐miRNA‐mRNA network.

The TFs of key genes were predicted by the JASPAR database and the ChEA database, of which BID (E2F1) and TNFAIP6 (CEBPB) predicted a TF, respectively (Figure 7A). In addition, the intersection of miRNAs was predicted by the TarBase and miRTarBase databases, and it was found that nine pairs of miRNA‐mRNAs intersected, including TMTC1, BID, and nine miRNAs (Figure 7B). After that, 225 miRNA‐targeting lncRNAs were predicted using the StarBase database (Figure 7C). The regulatory network of three mRNA‐two TF‐eight miRNA‐30 lncRNA was constructed through screening (Figure 7D). Among them, BID had a certain relationship with E2F1, hsa‐miR‐26b‐5p, and EBLN3P. In addition, The CTD database was used to predict 49 drugs corresponding to key genes, such as Paraquat, Valproic Acid, and bisphenol A (Figure 7E).

### 3.9 Key Genes Were Significantly Expressed in Nucleus Pulposus Cells

**FIGURE 8:**
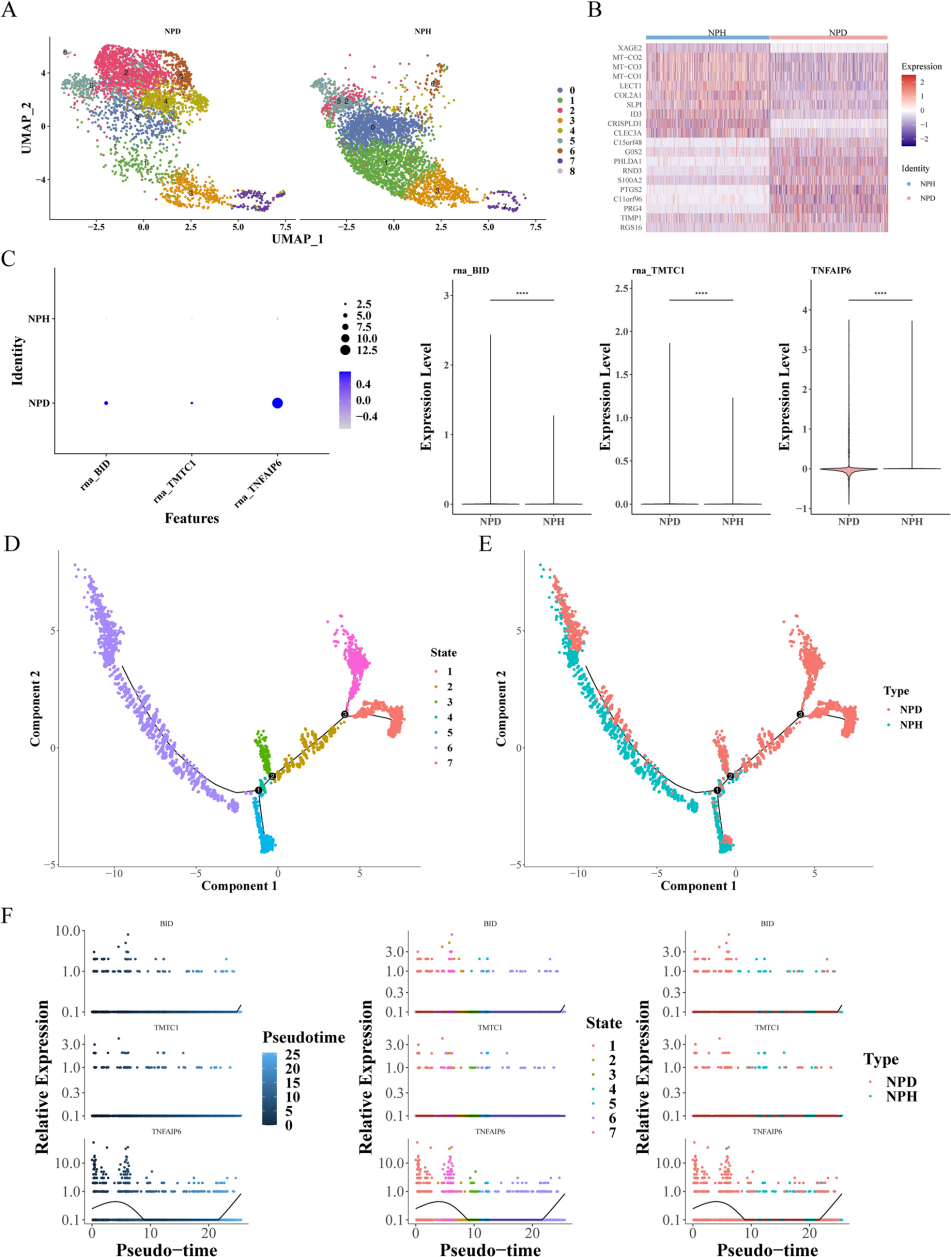
Single‐cell RNA‐sequencing (scRNA‐seq) analysis of the GSE199866 dataset. (A) Uniform manifold approximation and projection (UMAP) clustering analysis visualizing nine distinct cell clusters. The cells in the different cell clusters were all nucleus pulposus cells. (B) Heatmap displaying the distribution of marker genes in degraded and non‐degraded nucleus pulposus cell samples. (C) Expression profiles of key genes in degraded versus non‐degraded nucleus pulposus cell samples. (D, E) Pseudotime analysis of nucleus pulposus cells categorized into seven stages, illustrating their differentiation trajectories in both degraded and non‐degraded conditions. (F) Changes in the relative expression levels of key genes as nucleus pulposus cells mature through developmental stages. *p < 0.05, **p < 0.01, ***p < 0.001, ****p < 0.0001, ns: *p* > 0.05.

In order to detect the expression of key genes in single cells, we performed a single‐cell analysis. Firstly, 7471 cells and 19 284 genes were obtained after quality control of samples in GSE199866 (Figure S6). Then, the first 2000 highly variable genes were selected, and the resolution for cell group identification was set to 0.6, and the PCA number to 30 for subsequent analysis. The results showed that nine different cell populations were identified by UMAP cluster analysis (Figure 8A). Subsequently, 133 marker genes were identified in degraded and non‐degraded nuclei pulposus cell samples (Figure 8B). Then, the three key genes were significantly expressed between the two samples, and their expression levels were higher in the degraded samples than in the non‐degraded samples (Figure 8C). Finally, the cell trajectory differentiation of nucleus pulposus cells was simulated. The cells differentiated from right to left over time, and the cells had seven different differentiation states (Figure 8D), each marked with a different color, with red being the earliest type of differentiation, and the degeneration of nucleus pulposus cells from intervertebral disc to non‐degenerative differentiation (Figure 8E). Interestingly, the expression level of TNFAIP6 changed significantly during cell differentiation (Figure 8F). However, the expression of BID and TMTC1 did not change significantly.

## 4 Discussion

IDD impairs the disc's capacity to absorb shocks and maintain spinal flexibility, leading to pain and restricted movement [64, 65]. Despite existing evidence that somewhat elucidates the pathogenic mechanisms of IDD, research exploring the specific mechanisms from the perspective of mitochondrial dysfunction and PCD remains inadequate. This study bridges this gap by leveraging transcriptomic data and MR to screen and validate key genes related to PCD and mitochondrial function in IDD, further analyzing the biological pathways they are involved in. The results reveal that BID, TNFAIP6, and TMTC1 may have causal roles in IDD. We will explore the potential ramifications of these findings in the subsequent discussion.

BH3‐interacting domain death agonist (BID) is a protein‐encoding gene that can encode a member of the BCL‐2 family of cell death regulators and is also a mediator of mitochondrial damage induced by caspase‐8 [66]. BID plays an important role in both PCD and mitochondrial dysfunction, and it is considered a molecular link between mitochondrial function and various forms of peripheral cell death [67]. Simply put, the BH3 domain in BID interacts with Bcl‐2 family proteins to exert pro‐death effects, while cleaved BID can induce various mitochondrial dysfunctions, including reactive oxygen species production (ROS), cristae remodeling, intermembrane space content release, and permeability transition [67, 68]. According to research, the body regulates the degeneration of nucleus pulposus cells by managing the levels of BCL‐2, collagen, caspases, and proteoglycans. When BID expression increases, it undermines the protective role of BCL‐2, leading to a rise in apoptosis of notochordal cells within the nucleus pulposus, thus contributing to IDD. Furthermore, high BID expression might inhibit the anti‐inflammatory effects of BCL‐2 by diminishing the activation of the NLRP1 inflammasome and the release of IL1B, disrupting the metabolic balance of the ECM, intensifying local inflammatory responses in disc cells, and resulting in IDD [69]. This study's MR results also support a potential causal relationship between BID and IDD. Single‐cell analysis revealed that BID expression in nucleus pulposus cells is significantly higher in degenerated disc samples compared to non‐degenerated ones. Therefore, inhibiting BID expression may offer clinical benefits for IDD, and further research into targeted regulation of BID is warranted.

Tumor necrosis factor‐inducible protein 6 (TNFAIP6) is induced by tumor necrosis factor‐α (TNF‐α). Although TNF‐α itself is a pro‐inflammatory factor, TNFAIP6 not only plays a role in the inflammatory response but also participates in tissue repair and ECM stability [70]. Previously mentioned mechanical stress and local pressure are risk factors for IDD, and nucleus pulposus cells resist pressure by secreting ECM. Research has demonstrated that reducing TNFAIP6 expression leads to notable changes in both anabolic and catabolic genes associated with the ECM in nucleus pulposus cells [71]. TNFAIP6 may be involved in the stability of the ECM and cell migration through its binding to hyaluronic acid [72]. Additionally, increased levels of TNFAIP6, along with IL‐1 receptor antagonist, have been observed in inflammatory cells derived from the nucleus pulposus, annulus fibrosus, and endplate [73]. Recombinant human TSG‐6 (rhTSG‐6) is the laboratory‐produced form of the TNFAIP6 protein. In IL‐1β‐induced nucleus pulposus cell models, rhTSG‐6 was found to reduce ECM degradation and apoptosis by inhibiting multiple pathways [70]. Both in vitro and in vivo research has demonstrated that TSG‐6 decreases the expression of matrix metalloproteinases and inflammatory cytokines in degenerated nucleus pulposus tissue, thus exerting anti‐IDD effects [74]. MR results from this study define TNFAIP6 as a protective factor in IDD, which aligns with current research evidence. Previous studies have emphasized the relationship between TNFAIP6 and IDD in terms of its role in inflammation and the ECM. However, this study has identified the causal effect of TNFAIP6 on IDD through genetic, mitochondrial, and PCD pathways. Therefore, there may be additional potential roles of TNFAIP6 in IDD that have yet to be fully elucidated.

Transmembrane and tetratricopeptide repeat containing 1 (TMTC1) is a member of the TMTC family, which is part of the cadherin superfamily [75]. This family is associated with protein folding and stress responses within the endoplasmic reticulum (ER) and is involved in maintaining calcium homeostasis in the ER. Current evidence suggests TMTC1 is an integral membrane protein in the ER with multiple oriented TPR domains in the ER lumen. Overexpression of TMTC1 results in decreased calcium release from the ER upon stimulation, whereas knocking out TMTC1 leads to increased calcium release from the ER following stimulation [76]. The homeostasis of calcium ions in the ER is essential for various cellular functions such as protein folding, signal transduction, and enzyme activity [77, 78]. Disruption of calcium homeostasis can induce ER stress (ERS) [79]. ERS can stimulate the production of inflammatory cytokines such as tumor necrosis factor‐alpha (TNF‐α). Scholars have demonstrated that the employment of the pro‐inflammatory cytokine TNF‐α can lead to an upregulation in the expression of necroptosis‐related molecules (MLKL, and p‐MLKL RIPK1, p‐RIPK1, RIPK3, p‐RIPK3) within nucleus pulposus cells [80]. Furthermore, factors such as inflammation and Ca^2+^ imbalance leading to apoptosis and ECM degradation further advance IDD [81]. Importantly, ERS and mitochondrial dysfunction can collectively contribute to IDD through the interaction of various signaling pathways and key molecules like ROS [81]. Although there is no direct research exploring the correlation between TMTC1 and IDD, the role of TMTC1 in ER function and calcium regulation could be pivotal in the pathophysiological changes of IDD.

In the training set GSE70362, based on the expression levels of BID, TMTC1, and TNFAIP6, the presence or absence of IDD was used as the outcome event. Nomogram and clinical impact curves showed that these three targets have diagnostic value for IDD. GSEA enrichment analysis detailed the function of individual genes. BID is enriched in the apoptosis pathway, which is relevant to this study. A previous study has already presented evidence that BID leads to apoptosis of notochordal cells in the nucleus pulposus [69]. Cytokines and their receptors play a critical role in ECM remodeling, inflammatory responses, and intercellular communication, with a mutually dependent relationship between cytokines and ECM [82]. The enrichment of TNFAIP6 in the cytokine‐cytokine receptor interaction pathway suggests that TNFAIP6 may also regulate cytokines involved in the modulation of the ECM in IDD nucleus pulposus. Further immune infiltration analysis discovered that six immune cell types had higher enrichment scores in IDD samples compared to control samples, with TNFAIP6 showing a strong positive correlation with activated dendritic cells. During IDD, the immune environment shifts, causing activated dendritic cells to act as antigen‐presenting cells, which recognize and process injured disc tissue, thereby stimulating and modulating immune responses that increase local inflammation [83]. However, the exact molecular mechanisms regarding TNFAIP6 and dendritic cells in IDD are unknown and need to be explored further.

In this study, we constructed a TF‐mRNA‐miRNA‐lncRNA network and discovered complex regulatory mechanisms. Notably, we found that the key gene BID is related to miR‐26a‐5p. miR‐26a‐5p is thought to exacerbate the development of IDD by targeting the Smad1 signaling pathway [84]. The potential mechanisms involving miR‐26a‐5p, BID, and IDD identified in this study may yield clinical benefits. Using the CTD database, we predicted the corresponding chemicals for three genes (BID, TMTC1, TNFAIP6). Among the highly correlated chemicals are troglitazone, valproic acid, and quercetin. Currently, troglitazone is used for the treatment of type 2 diabetes mellitus, while valproic acid has antiepileptic effects. However, there is no evidence supporting the use of either drug for IDD. Quercetin is found in various traditional Chinese medicines, and network pharmacology reports indicate that it is an active ingredient in multiple traditional Chinese medicine formulations [85, 86]. Therefore, further investigation into drugs that regulate the targets of this study, specifically those containing quercetin, is warranted.

## 5 Conclusion

This study identifies potential causal evidence for the involvement of PCD and mitochondrial‐related genes in IDD, providing a theoretical basis for further comprehensive elucidation of IDD pathogenesis and targeted therapy, which holds certain clinical significance. However, the study has the following limitations: (1) In the MR analysis, we used PCD and mitochondrial‐related genes as exposure factors and IDD as the outcome factor. The IVs for the target genes were assumed to have a lifelong impact on IDD, but actual levels of target proteins can vary in the human body, possibly biasing the study's conclusions. (2) The IDD data originates from a European population; hence utilizing the results for other populations may introduce some bias. (3) Further wet‐lab experiments are needed to validate the identified targets, but we will continue to monitor these targets.

## Author Contributions

All authors contributed to the study. The first draft of the manuscript was written by Hongfei Nie, and all authors commented on previous versions of the manuscript. All authors read and approved the final manuscript.

## Ethics Statement

This study was performed in line with the principles of the Declaration of Helsinki. Approval was granted by the Ethics Committee on Biomedical Research, West China Hospital of Sichuan University.

## Consent

The authors have nothing to report.

## Conflicts of Interest

The authors declare no conflicts of interest.

## Supporting information


**Fig. S1.** Sample clustering diagram from GSE70362 dataset among WGCNA. The original analysis identified GSM1725809 as an outlier, which was subsequently removed. The figure presented here includes the clustering results before the removal of GSM1725809.

**Fig. S2.** Scatter plot from MR analysis for causal associations between candidate genes and IDD.

**Fig. S3.** Forest plot from MR analysis for causal associations between candidate genes and IDD.

**Fig. S4.** Funnel plot from MR analysis for causal associations between candidate genes and IDD.

**Fig. S5.** The forest plot of leave‐one‐out (LOO) test for sensitivity analysis of candidate genes.

**Fig. S6.** The metrics of nFeature_RNA, nCount_RNA, and percent_mt before (up) and after (down) quality control (QC) in GSE199866 dataset.

**Tables S1–S3.** Sensitivity analysis methods confirm the reliability of MR analysis. This table includes outcomes from the Steiger test and assessments of heterogeneity and pleiotropy in genes implicated in the causality of IDD.

## Data Availability

The datasets (GSE70362, GSE56081 and GSE199866) analyzed in this study can be found in the Gene Expression Omnibus (GEO) database (https://www.ncbi.nlm.nih.gov/gds). The dataset used for MR analysis (ukb‐b‐19 807) is available for download from the IEU OpenGWAS database (https://gwas.mrcieu.ac.uk). The 1136 MRGs can be accessed from the MitoCarta 3.0 database (https://www.broadinstitute.org/mitocarta). Additionally, the 1548 PCDRGs can be obtained from previously published literature.
