# Supplementary figures and images for: Transcriptome Data Combined With Mendelian Randomization Analysis Identifies Key Genes Associated With Mitochondria and Programmed Cell Death in Intervertebral Disc Degeneration
Source: JOR Spine. 2025 Mar 24;8(1):e70057. doi: 10.1002/jsp2.70057 (PMC11931668; doi:10.1002/jsp2.70057)

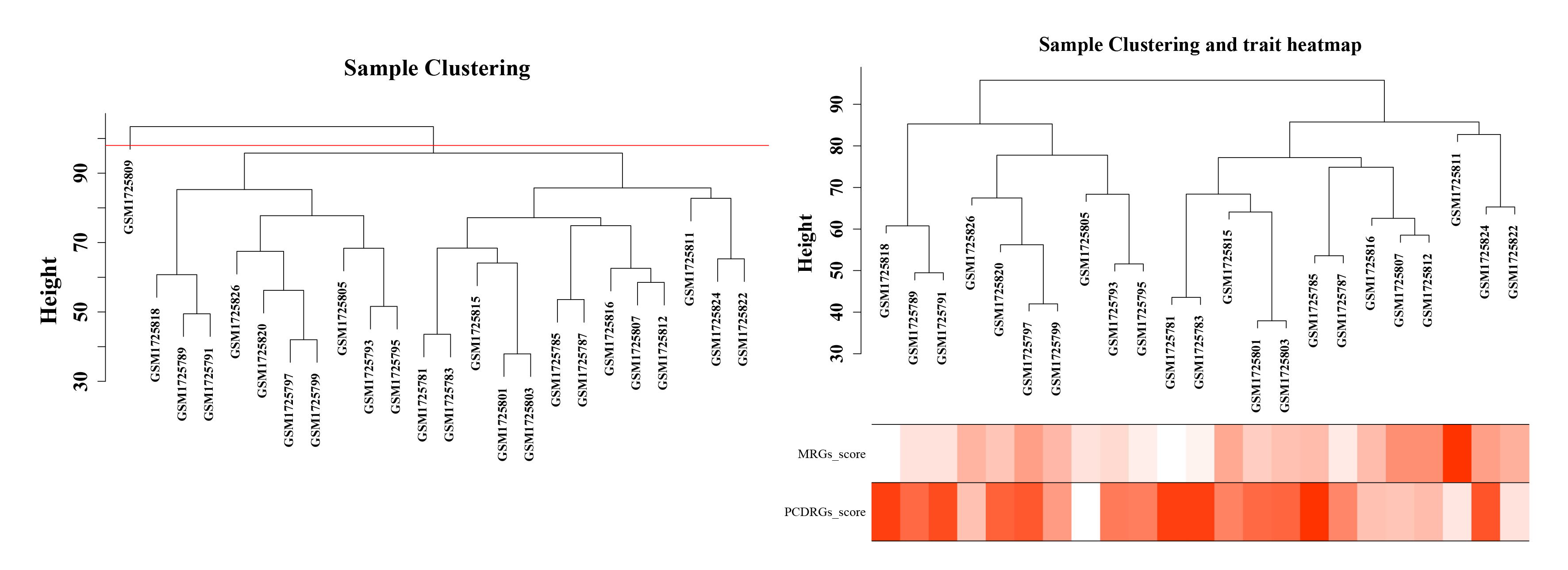

Supplement: Supplementary file 1 — Fig. S1. Sample clustering diagram from GSE70362 dataset among WGCNA. The original analysis identified GSM1725809 as an outlier, which was subsequently removed. The figure presented here includes the clustering results before the removal of GSM1725809. [file JSP2-8-e70057-s002.tif]

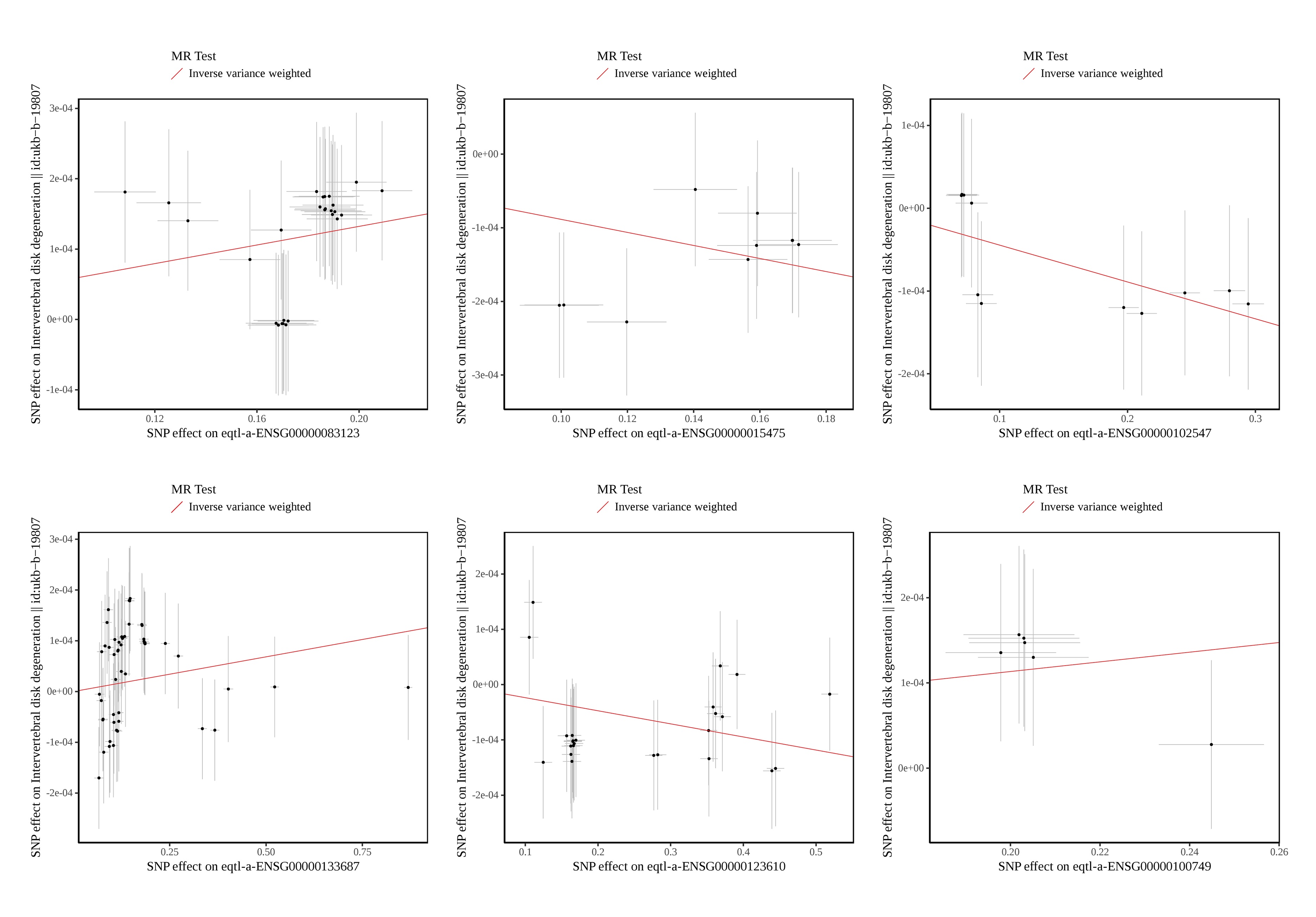

Supplement: Supplementary file 2 — Fig. S2. Scatter plot from MR analysis for causal associations between candidate genes and IDD. [file JSP2-8-e70057-s005.jpg]

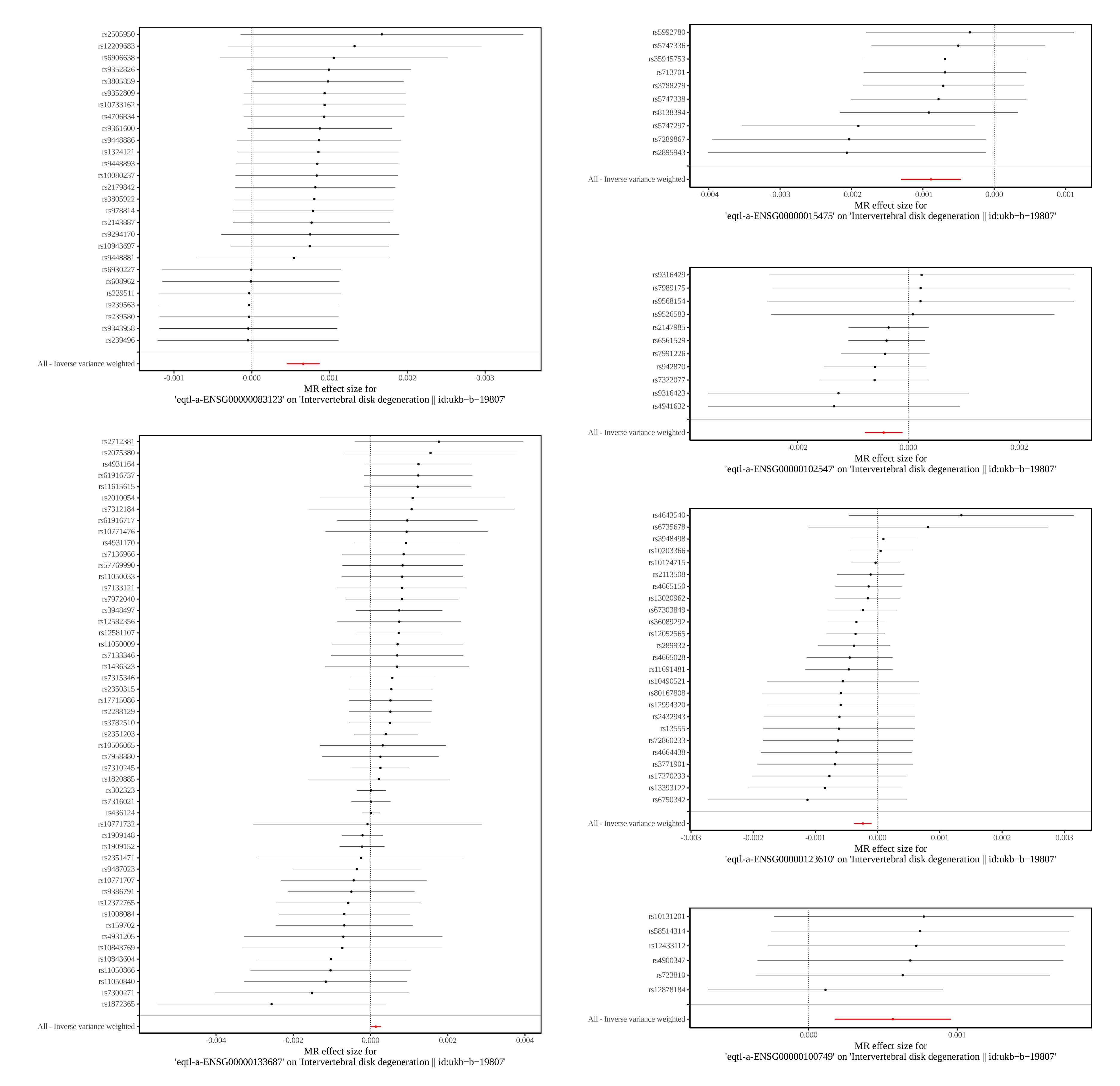

Supplement: Supplementary file 3 — Fig. S3. Forest plot from MR analysis for causal associations between candidate genes and IDD. [file JSP2-8-e70057-s004.jpg]

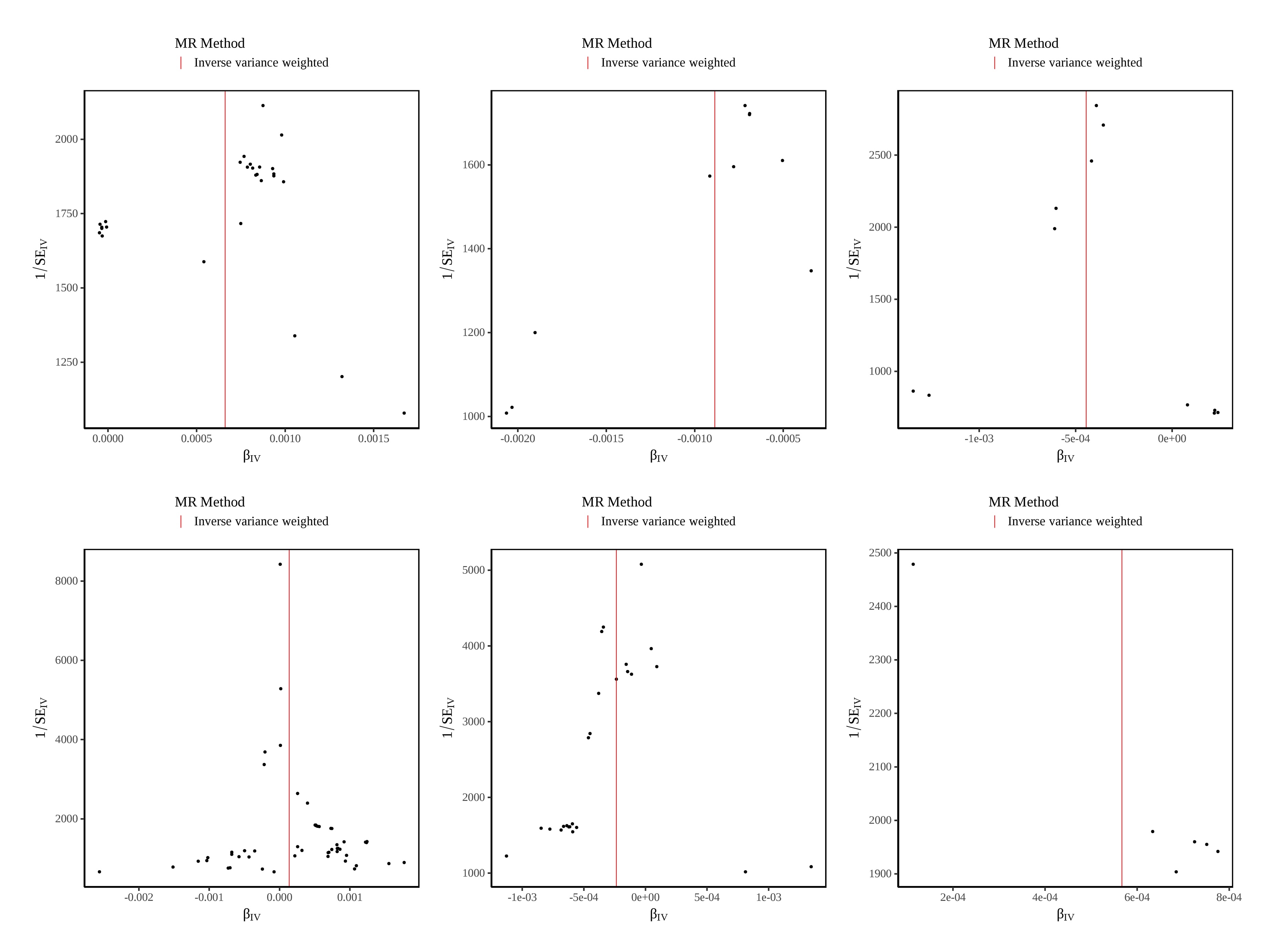

Supplement: Supplementary file 4 — Fig. S4. Funnel plot from MR analysis for causal associations between candidate genes and IDD. [file JSP2-8-e70057-s006.jpg]

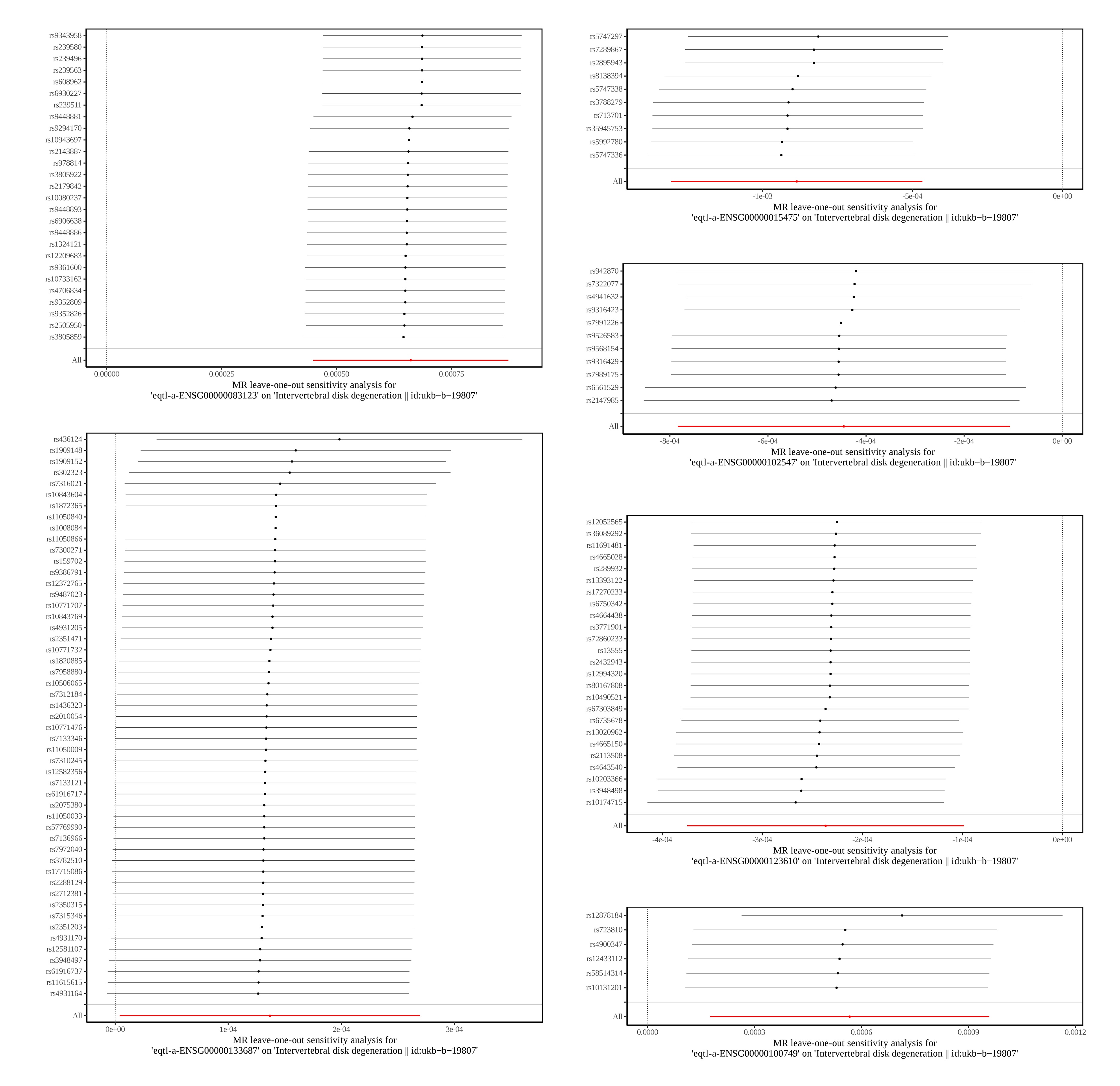

Supplement: Supplementary file 5 — Fig. S5. The forest plot of leave‐one‐out (LOO) test for sensitivity analysis of candidate genes. [file JSP2-8-e70057-s003.jpg]

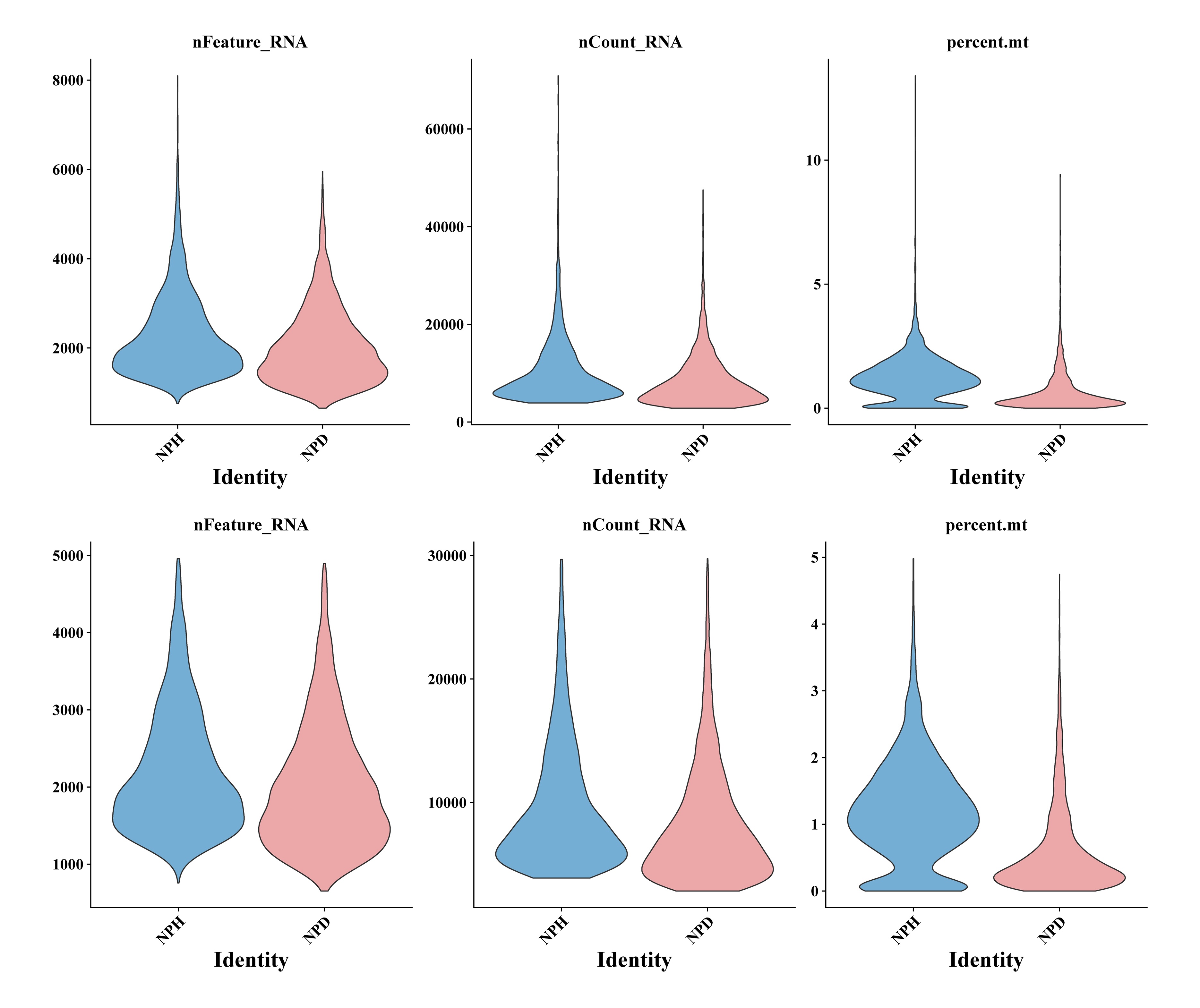

Supplement: Supplementary file 6 — Fig. S6. The metrics of nFeature_RNA, nCount_RNA, and percent_mt before (up) and after (down) quality control (QC) in GSE199866 dataset. [file JSP2-8-e70057-s001.jpg]
